# What to Do When There Is Something Unexpected?

**DOI:** 10.3390/life14020213

**Published:** 2024-01-31

**Authors:** Vlad Sabin Ivan, Daniel-Florin Lighezan, Melania Ardelean, Nicoleta Balteș, Alexandra Corina Faur, Paul-Gabriel Ciubotaru, Adina-Flavia Cutina-Morgovan, Roxana Buzaș

**Affiliations:** 1 Department of Internal Medicine I, “Victor Babeș” University of Medicine and Pharmacy, 300041 Timișoara, Romania; vlad_sabin_ivan@yahoo.com (V.S.I.); dlighezan@umft.ro (D.-F.L.); ardelean.melania@gmail.com (M.A.); adina.morgovan@yahoo.com (A.-F.C.-M.); roxanabuzas@yahoo.com (R.B.); 2 Center for Advanced Research in Cardiovascular Pathology and Hemostaseology, “Victor Babeș” University of Medicine and Pharmacy, 300041 Timișoara, Romania; 3 Gastroenterology Unit, Emergency Clinical Municipal Hospital, 300079 Timișoara, Romania; baltesnicoleta@yahoo.ro; 4 Department of Anatomy and Embriology, “Victor Babeș” University of Medicine and Pharmacy, 300041 Timișoara, Romania; alexandra_pantu@yahoo.com

**Keywords:** digestive hemorrhage, neoplasia, myocardial infarction, rectal cancer

## Abstract

Background: Myocardial infarction is currently the leading cause of death worldwide, followed by malignant neoplasms. The presence of both within the same patient obviously increases the risk of death, as many coronary events are detected in patients diagnosed with cancer. Diagnosis of an occult digestive cancer in the acute phase of myocardial infarction is most frequently prompted by a hemorrhagic complication. Case summary: This case features an 81-year-old male patient diagnosed with acute myocardial infarction, treated with primary percutaneous intervention (PCI), who developed post-stenting hemorrhagic complications in the first 24 h due to the presence of two different concomitant malignant neoplasms. The outcome was favorable in the acute phase, even if de-escalation therapy was given immediately post-stenting, and intrastent residual thrombotic risk was high. Conclusions: The presence of bleeding complications in patients with acute myocardial infarction should mobilize resources in search of a neoplastic cause, especially a digestive one. However, other locations should be looked for, depending on the source of bleeding.

## 1. Introduction

Patients with malignant neoplasia have an increased risk of cardiovascular events, with the two clinical entities interrelating in a complex way. The therapy of one of them influences the evolution, complications, and prognosis of the other, in a bidirectional relationship. Patients with malignant neoplasia have been excluded from most clinical trials investigating cardiovascular events for several reasons. The first of these is mainly related to possible short survival and possible bias regarding associated cardiac events, as well as to ethical reasons [1].

The proportion of patients with acute coronary syndromes who are already diagnosed with cancer, or are diagnosed immediately after an acute coronary event, varies between 3% and 17% [2,3].

The pathophysiological mechanism that can trigger a thrombotic event in a patient with neoplasia, regardless of location, is a complex mechanism involving both classical factors and those resulting from the action of neoplasia, such as anemia, hypervascularity, or increased oxygen consumption, to which pro-coagulant factors are associated with an induced hypercoagulopathy [4].

Activation of lymphocytes, with increased synthesis of interleukin 1 and 6, tumor necrosis factor, and the monocyte-macrophage system, are involved in the abnormal activation of the coagulation cascade through activation of platelets, endothelial adhesion, and endothelial growth factors [5].

Treatment of these patients is often problematic, with the current recommendations being of a general nature, and should be individualized to avoid complications [6]. The incidence of acute coronary syndromes is highest within 6 months of a newly diagnosed cancer, being 66% higher than in the general population [7].

Although, as mentioned above, most occurrences are reported in cases already diagnosed with neoplasia, and data in the literature are very limited regarding the detection of acute coronary artery disease.

## 2. Case Presentation

**Figure 1:**
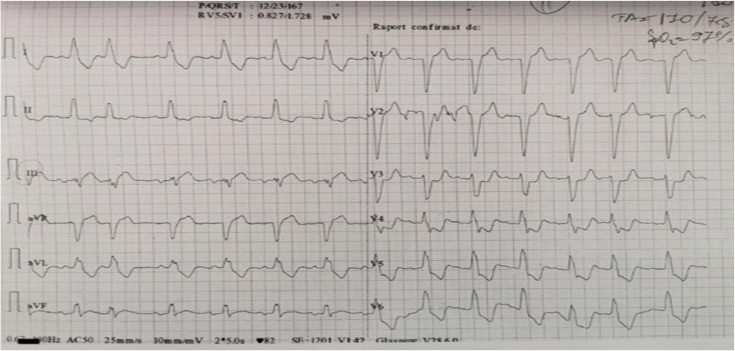
EKG at presentation (standard calibration at 82 b/min—2 instances of 5 s).

The patient is an 81-year-old male who is a former smoker with about 30 packs/year, and a non-smoker for about 15 years who has a known familial history of colorectal cancer. No prior cancer screening was performed on the patient. His personal history includes arterial hypertension and dyslipidemia, with intermittent episodes of stable angina pectoris at high exertion and prostatic hyperplasia. At 06:00 a.m. the patient presented at the Emergency Department after several intensive angina pectoris episodes that partially succumbed to administration of sublingual nitroglycerin, which started 2 h before arrival. Physical examination on admission revealed a BMI of 29.5 kg/m^2^ SC, and a BP of 170/75 mmHg, with no other significant pathological features detectable at the time of presentation. The EKG showed a sinus rhythm with left bundle branch block (age undetermined) (Figure 1); high-sensitivity cardiac troponin (hs-cTn) levels at presentation were normal, with a CK-MB above normal; and 1 h later, hs-cTn showed an increase of more than 10 times the normal value.

Blood chemistry showed a moderate normochromic normocytic anemia (9.8 g/dL), with a serum creatinine of 1.58 mg/dL. The Nt-proBNP was 631 pg/mL, and ferritin was 29.8 ng/mL, with the lower value of normal being 21 ng/mL as per local laboratory.

The transthoracic echocardiography revealed altered global and segmental functions with an LVEF of 44% with mild mitral regurgitation. Speckle tracking indicated severe hypokinesis to akinesis in the inferior and posterior segments of the left ventricle. Chronic medication included perindopril 5 mg od, atorvastatin 20 mg od, aspirin 75 mg od, tartrate metoprolol 25 mg bid, and nitroglycerin during anginal episodes.

**Figure 2:**
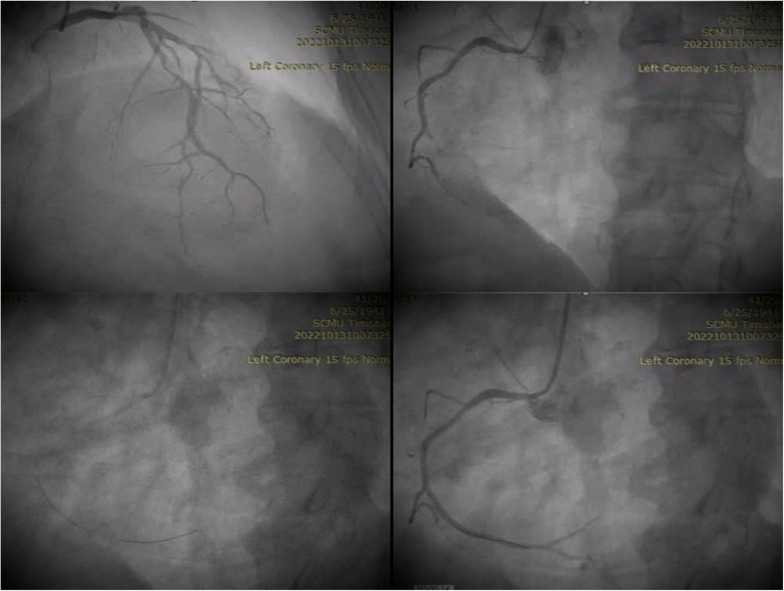
Coronarography.

A coronary angiogram was performed, which revealed the trunk of the left coronary artery, circumflex artery, and atheromatous anterior descending artery, but without occlusions (Figure 2, top left). However, an occlusion on the right coronary artery was identified (Figure 2, top right image), and a PTCA was performed on this artery (Figure 2, bottom left image) with the angiographic result TIMI 3 (Figure 2, bottom right).

The immediate post-stenting evolution was favorable under anticoagulant treatment and double antiplatelet therapy. At 12 h post-procedure, the patient presented with hematochezia and massive hematuria, with hemoglobin levels decreasing by 2.5 g/dL; clopidogrel de-escalation was performed.

**Figure 3:**
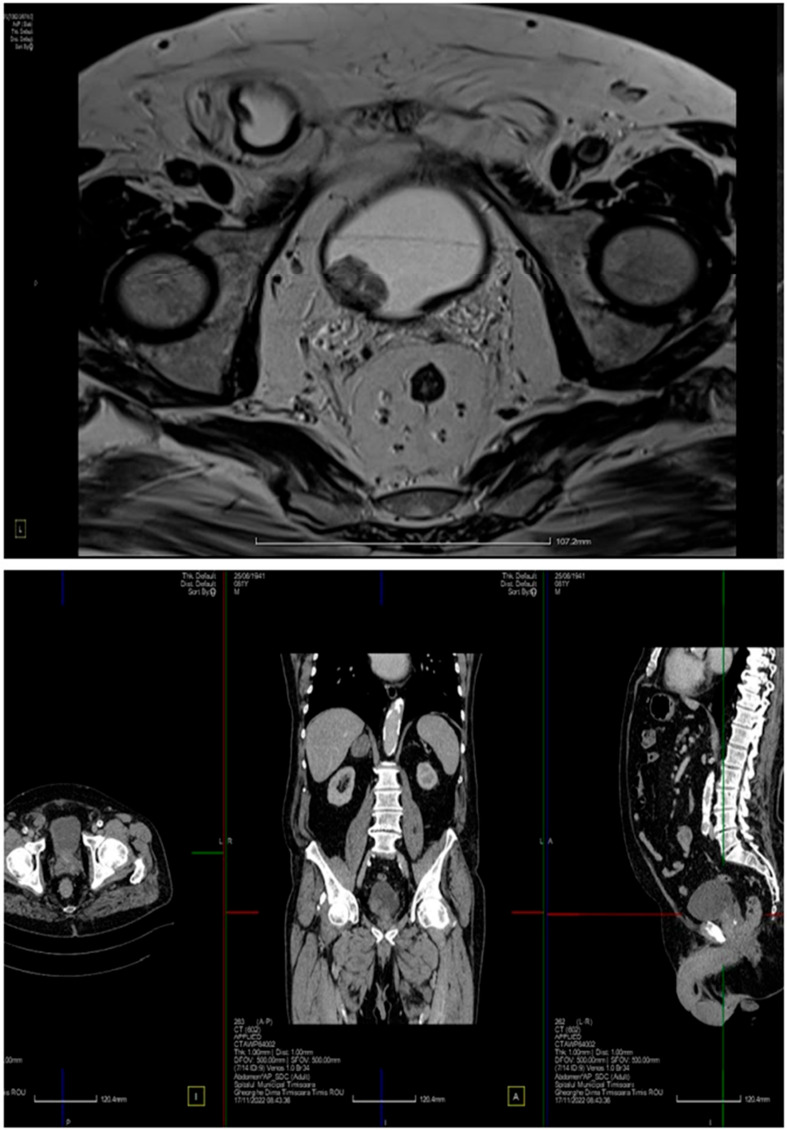
MRI imaging (**upper**), and abdominal and pelvic CT (**lower**).

On rectal examination, a tumor formation was identified approximately 5 cm from the external anal opening, and a recto-sigmoidoscopy with biopsy sampling was performed. Imaging (Figure 3) suggested the presence of both a rectal tumor and the presence of an intravesical tumor. The computed tomography showed a diffuse thickening of the lower rectum at 6 cm in length, just above the anal canal, with bilateral infracentimetric inguinal lymph nodes. Other significant elements of the digestive tract were a small inguinal uncomplicated hernia and several diverticula of the sigma. On the other hand, the tomography identified a suspicious mass (30 mm in diameter) on the lateral wall of the bladder and a protruding median lobe of the prostate.

Because of this finding, a second high performance imaging was recommended, and an MRI was scheduled. The MRI showed a mass that involved the tunica muscularis of the rectum above the anal canal and perirectal lymph nodes, using T2 and DWI imaging. The mass in the bladder was characterized as a tumor limited to the mucosa, with no visible local lymph nodes, and with isointense T1 and hyperintense T2 signals.

Under de-escalation therapy and conservative therapy, the evolution was towards remission of the bleeding episode. The treatment with clopidogrel was withdrawn and the next dose after the first bleeding event was not administered; following this, the patient was moved to the ICU for monitoring because of the risks associated with stent thrombosis. The anticoagulant therapy was reduced to 4000 IU bid of enoxaparine as the patient was receiving 8000 IU subcutaneous bid of enoxaparine at that time. Aspirin was maintained at a standard dose, as per protocol, of 75 mg od.

The patient’s course was favorable, and the patient was discharged after 12 days.

Three months after PCI, surgery was performed with the intention of radicality, with a left iliac colostomy and resection of the lower sigma. This was decided by the surgery team together with the patient, based on the presence of multiple diverticula of the lower sigma and the risk of inflammation during subsequent treatments.

The final histopathological result was a pT3N1Mx adenocarcinoma. The workflow protocol for those samples were as follows: the tissue samples were fixed in formalin (10% dilution), paraffin-embedded, and sectioned. The 4 µ-thick, formalin-fixed, paraffin-embedded tissue samples were stained with hematoxylin and eosin (HE). We used a Leica DM750 microscope with a digital camera for images acquisition, with magnification of 20× and 40× objectives (×20 Ob, ×40 Ob). Microscopic examination revealed in the samples collected from the exophytic lesion a moderately differentiated gland forming carcinoma with neoplastic glands filled with necrotic debris and desmoplasia. A diagnosis of a moderately differentiated adenocarcinoma was formulated. The small polipoid area of the mucosa showed the microscopic features of a tubulovillous adenoma. The second described mass was also approached intravesically by TUR-V with endoscopic resection during the second part of surgery, one month after rectal resection, also resulting in pT2NxMx urothelial adenocarcinoma.

## 3. Discussion

The diagnosis of malignant neoplasia in acute coronary syndrome is unfortunately not unique, but having two different neoplasia at the same time in the presence of acute coronary syndrome is problematic. Studies report incidences of this ranging from 1–2% to 30%. The diagnosis is usually established within the first 6 months of an acute coronary event [8], even more so as the cancer disease is in the metastatic phase. The most common malignant neoplasms involved are pulmonary, gastric, or pancreatic.

A more common presentation of acute coronary syndrome is in the form of a non-STEMI infarction, with silent ischemia being more frequent. Its presentation is often atypical, masquerading as dyspnea, pain is relieved by the prescription of analgesics, and the proportion of type 2 myocardial infarction is higher in up to a quarter of cases [9]. Patients with type 2 infarction usually have more advanced cancer, have additional comorbidities, or are receiving chemotherapy.

It is a significant issue that many cancer patients have been excluded from clinical trials that have assessed the impact of medication. Consequently, there is a scarcity of data in the literature, and much of the information is based on consensus. As a result of this problem, treatment guidelines do not offer explicit recommendations based on concrete evidence, and it is usually personalized to suit individual needs [10]. Furthermore, most patients with an oncological disease do not receive optimal therapy, with only one third of patients receiving optimal treatment [11]. A study published by Yusuf et al. [12] showed a doubling of post-infarction survival in cancer patients who received aspirin (36% vs. 18%), although only 46% of these were patients with myocardial infarction, of which 15% with STEMI received aspirin. This fact suggests a major discrepancy between generalizing treatment guidelines and special populations, such as neoplastic patients.

In terms of peri-event diagnosis, data are even scarcer. Approximately 10% of patients in one study who also had PCI [13] were found to have neoplasia, 3% of patients were on active antineoplastic therapy, non-STEMI, and unstable angina was present in approximately 70% of presentations. Of the antithrombotic treatment, 51% received clopidogrel, 39% received ticagrelor, and 10% received prasugrel.

Another study [14] suggests a rate of one newly diagnosed cancer for every thirteen cases of bleeding in patients on newly instituted antiplatelet therapy for an acute coronary syndrome, most commonly located in the genitourinary and digestive tracts. A bleeding event makes the probability of being diagnosed with neoplasia after PCI and double antiplatelet therapy 3.6 times higher than in the absence of bleeding, with a positive predictive value of neoplasia diagnosis of 7.7%. The conclusion of this study is that bleeding after stenting in the first 6 months and optimal medical therapy are associated with the presence of neoplasia that was not evident at the time of PCI.

A similar result [15] from a population-based analysis including 2.5 million patients with an average follow-up rate of 7.3 years showed that the use of at least one antiplatelet in combination or not with an anticoagulant suggested that the occurrence of hematuria within the first 6 months of institution was associated with urothelial neoplasia, the incidence being almost double that of those who did not take an antiplatelet or were diagnosed at more than 6 months.

On the other hand, the peculiarity of this case is the concomitant existence of two different neoplasia, this being rather uncommon. There are few studies that evaluate such cases. In a cohort of more than 2 million cancer patients, 8.1% of survivors developed a second malignancy [16]. The majority of such cases occurred after the treatment of a first malignant neoplasm of hematological origin [17].

Another feature of this case is also the onset of a digestive bleeding immediately after initiation of the specific post-PCI therapy, which revealed the presence of a rectal neoplasia of the adenocarcinoma type, which concomitantly presents a clinically silent intravesical tumor formation, with the appearance of urothelial neoplasia. Additionally, an immediate post-stenting bleeding event raises outcome and prognostic issues with the need for discontinuation of dual antiplatelet therapy, with underlying risks, while clinical judgment and the balance between in-stent thrombotic risk and major bleeding risk are probably most important for the evolution and improvement of symptoms.

This case highlights the importance of thorough clinical examination before any medical or surgical intervention, if the clinical status allows. The presence of two different cancers in a patient who recently received a coronary angiography with stent placement is a ticking time bomb for bleeding, especially in the digestive and genito-urinary systems. For example, if the CRUSADE score had been taken into account at admission, only some variables and some key biochemical characteristics would have been used as a diagnostic tool. CRUSADE score was developed as a tool for quantifying bleeding risk for in-hospital major bleeding in the presence of a NSTEMI. This score includes several parameters that were validated through other studies especially in patients who receive multiple antithrombotics. The mentioned score for this patient, if it had been calculated, would have shown an 8.2% risk of bleeding, which would have been very high, and probably would have warranted a cautionary approach to administering higher doses of anticoagulants and antiplatelets. It is true, based on this score, that the higher the risk of bleeding the better the outcomes of those patients because the thrombotic risk is even higher, and they benefit from it [18].

In elderly patients, invasive procedures are associated with an increase in risk for certain complications, but recent data suggest that an early invasive approach in patients with myocardial infarction reduces overall mortality. The cardio CHUS-HUSJ registry showed a reduction in overall mortality, even though this is a retrospective study [19]. Another study that included over 2300 patients with a median age of 86 years showed that the invasive approach was associated with improved survival, even in patients with high frailty indexes Among low, medium, and high frailty subgroups with adjusted HRs of 0.74 (95% CI, 0.58 to 0.93), 0.65 (95% CI, 0.50 to 0.85), and 0.52 (95% CI, 0.34 to 0.78), respectively, (P for interaction = 0.48) the benefit was consistent. [20].

Patients with active or past cancer presenting to the ED with acute chest pain had about 1.5 times the prevalence of a final adjudicated diagnosis of NSTEMI, compared to patients without cancer. Tachycardia remained the most frequent type 2 NSTEMI trigger, although anemia was more frequent in patients with cancer than in those without. In our cohort, a greater proportion of patients with cancer were treated with long-term cardio-vascular medications at presentation compared to those without cancer [21].

Due to the pandemic, numerous patients are experiencing delays in receiving a proper diagnosis. As a result, an acute illness, such as a small myocardial infarction that has minor repercussions on overall health, may reveal a more severe diagnosis. Dyspnea and low oxygen saturation are evaluated extensively in the emergency room to the detriment of other symptoms that are often neglected [22,23].

## 4. Conclusions

In conclusion, in an elderly patient that presents with a picture of myocardial infarction, the clinician should bear in mind that there is a possibility of an occult cancer, especially in the setting of a non-STEMI, and the presence of two distinct cancers should not be a surprise.

## Data Availability

Data is contained within the article.

## References

[1] Lucà F., Parrini I., Abrignani M.G., Rao C.M., Piccioni L., Di Fusco S.A., Ceravolo R., Bisceglia I., Riccio C., Gelsomino S. (2022). Management of Acute Coronary Syndrome in Cancer Patients: It’s High Time We Dealt with It. J. Clin. Med..

[2] Rohrmann S., Witassek F., Erne P., Rickli H., Radovanovic D. (2018). Treatment of Patients with Myocardial Infarction Depends on History of Cancer. Eur. Heart J. Acute Cardiovasc. Care.

[3] Wang F., Gulati R., Lennon R.J., Lewis B.R., Park J., Sandhu G.S., Wright R.S., Lerman A., Herrmann J. (2016). Cancer History Portends Worse Acute and Long-term Noncardiac (but Not Cardiac) Mortality after Primary Percutaneous Coronary Intervention for Acute ST-Segment Elevation Myocardial Infarction. Mayo Clin. Proc..

[4] Libby P., Kobold S. (2019). Inflammation: A Common Contributor to Cancer, Aging, and Cardiovascular Diseases—Expandingthe Concept of Cardio-Oncology. Cardiovasc. Res..

[5] Mitrugno A., Tormoen G.W., Kuhn P., McCarty O.J. (2016). The Prothrombotic Activity of Cancer Cells in the Circulation. Blood Rev..

[6] Caine G.J., Stonelake P.S., Lip G.Y.H., Kehoe S.T. (2002). The Hypercoagulable State of Malignancy: Pathogenesis and Current Debate. Neoplasia.

[7] Mrotzek S.M., Lena A., Hadzibegovic S., Ludwig R., Al-Rashid F., Mahabadi A.A., Mincu R.I., Michel L., Johannsen L., Hinrichs L. (2021). Assessment of coronary artery disease during hospitalization for cancer treatment. Clin. Res. Cardiol..

[8] Radmilovic J., Di Vilio A., D’andrea A., Pastore F., Forni A., Desiderio A., Ragni M., Quaranta G., Cimmino G., Russo V. (2020). The Pharmacological Approach to Oncologic Patients with Acute Coronary Syndrome. J. Clin. Med..

[9] Milazzo V., Cosentino N., Campodonico J., Lucci C., Cardinale D., Cipolla C.M., Marenzi G. (2020). Characteristics, Management, and Outcomes of Acute Coronary Syndrome Patients with Cancer. J. Clin. Med..

[10] Zaleska M., Mozenska O., Bil J. (2018). Statins Use and Cancer: An Update. Futur. Oncol..

[11] Iannaccone M., D’Ascenzo F., Vadalà P., Wilton S.B., Noussan P., Colombo F., Raposeiras-Roubín S., Abu-Assi E., González-Juanatey J.R., Henriques J.P.S. (2017). Prevalence and Outcome of Patients with Cancer and Acute Coronary Syndrome Undergoing Percutaneous Coronary Intervention: A BleeMACSSub-study. Eur. Heart J. Acute Cardiovasc. Care.

[12] Yusuf S.W., Daraban N., Abbasi N., Lei X., Durand J.-B., Daher I.N. (2012). Treatment and Outcomes of Acute Coronary Syndrome in the Cancer Population. Clin. Cardiol..

[13] Pravecek M.K.P.M.K., Miskic B.M.B., Lukenda K.C.L.K.C. (2023). Cancer and Acute Coronary Syndrome. Eur. J. Prev. Cardiol..

[14] Raposeiras-Roubín S., Abu-Assi E., Muñoz-Pousa I., Rossello X., Cespón-Fernández M., Viu M.M., Caneiro-Queija B., Cobas-Paz R., Bastos G., Iñíguez-Romo A. (2020). Usefulness of Bleeding after Acute Coronary Syndromes for Unmasking Silent Cancer. Am. J. Cardiol..

[15] Wallis C.J.D., Juvet T., Lee Y., Matta R., Herschorn S., Kodama R., Kulkarni G.S., Satkunasivam R., Geerts W., McLeod A. (2017). Association between Use of Antithrombotic Medication and Hematuria-Related Complications. JAMA.

[16] Donin N., Filson C., Drakaki A., Tan H., Castillo A., Kwan L., Litwin M., Chamie K. (2016). Risk of second primary malignancies among cancer survivors in the United States, 1992 through 2008. Cancer.

[17] Munker R., Hiller E., Melnyk A., Gutjahr P. (1996). Second malignancies. Int. J. Oncol..

[18] Subherwal S., Bach R.G., Chen A.Y., Gage B.F., Rao S.V., Newby L.K., Wang T.Y., Gibler W.B., Ohman E.M., Roe M.T. (2009). Baseline risk of major bleeding in non-ST-segment-elevation myocardial infarction: The CRUSADE (Can Rapid risk stratification of Unstable angina patients Suppress ADverse outcomes with Early implementation of the ACC/AHA Guidelines) Bleeding Score. Circulation.

[19] Ferrero T.G., Álvarez B., Cordero A., Martínez J.M., Antonio C.C., Sestayo-Fernández M., Bouzas-Cruz N., Muiños P.A., Casas C.A.J., García O. (2022). Early angiography in elderly patients with non-ST-segment elevation acute coronary syndrome: The cardio CHUS-HUSJ registry. Int. J. Cardiol..

[20] Fishman B., Sharon A., Itelman E., Tsur A.M., Fefer P., Barbash I.M., Segev A., Matetzky S., Guetta V., Grossman E. (2022). Invasive Management in Older Adults (n elderly patients with non-ST-segment elevation acute coronary syndrome. Mayo Clin. Proc..

[21] Shobayo F., Bajwa M., Koutroumpakis E., Hassan S.A., Palaskas N.L., Iliescu C., Abe J.-I., Mouhayar E., Karimzad K., Thompson K.A. (2022). Acute coronary syndrome in patients with cancer. Expert Rev. Cardiovasc. Ther..

[22] Bima P., Lopez-Ayala P., Koechlin L., Boeddinghaus J., Nestelberger T., Okamura B., Muench-Gerber T.S., Sanzone A., Skolozubova D., Djurdjevic D. (2023). Chest Pain in Cancer Patients: Prevalence of Myocardial Infarction and Performance of High-Sensitivity Cardiac Troponins. JACC CardioOncol..

[23] Freund O., Azolai L., Sror N., Zeeman I., Kozlovsky T., Greenberg S.A., Epstein Weiss T., Bornstein G., Tchebiner J.Z., Frydman S. (2023). Diagnostic delays among COVID-19 patients with a second concurrent diagnosis. J. Hosp. Med..

